# Parents’ perceived barriers and enablers to providing optimal infant oral care

**DOI:** 10.1186/s12889-025-22487-9

**Published:** 2025-04-05

**Authors:** Olivia Walsh, Amrit Chauhan, My-Van Trinh, Clare Lin, Sarah Marshall, Kara A Gray-Burrows, Mihiri Silva

**Affiliations:** 1 Inflammatory Origins, Murdoch Children’s Research Institute, Royal Children’s Hospital, 70 Flemington Rd, Parkville, VIC 3052 Australia; 2 School of Dentistry, University of Leeds, Clarendon Way, Leeds, LS2 9JT UK; 3 Melbourne Dental School, University of Melbourne, 720 Swanston St, Carlton, 3053 VIC Australia; 4 Dental Health Services Victoria, 720 Swanston St, Carlton, 3053 VIC Australia; 5 Institute for Physical Activity and Nutrition, School of Exercise and Nutrition Science, Deakin university, Geelong, VIC 3220 Australia

**Keywords:** (3–10): oral health, Behaviour change, Pediatric dentistry, Infant care, Child, Toothbrushing

## Abstract

**Background:**

Early childhood caries (tooth decay) can adversely affect child growth, development and well-being and is a leading cause of preventable hospitalisation for pre-school aged children. This necessitates the introduction of preventive measures in infancy, including twice daily toothbrushing and timely dental visits. This study explored the barriers and enablers parents face in providing optimal oral care for their young children.

**Methods:**

We interviewed Australian parents with 0-36-month-old children about two key behaviours related to their child’s oral health: (1) the timing of first dental visit and (2) twice daily toothbrushing. Parents were recruited via social media advertising and all interviews were conducted online via Zoom. Interviews were based on a semi-structured interview guide mapped to the Theoretical Domains Framework (TDF). All interviews were audio recorded and transcribed. Data was coded to the TDF, summarised, and categorised as a barrier or enabler before being grouped into themes and sub-themes using framework analysis.

**Results:**

Fifteen interviews were completed between May 2022– May 2023. Thirteen of the 14 TDF domains were represented in the data. The three most dominant TDF domains across the dataset were *social influences*,* environmental context and resources*, and *knowledge.* Four themes were developed from the data: (1) Conflict, (2) Family and social norms, (3) Wanting a positive oral health experience, and (4) Uncertainty. These themes represent both barriers and enablers to optimal infant and young children’s oral care. Parents face complex decision-making challenges regarding their young children’s oral health care, particularly managing actual and perceived conflicts with their child. Knowledge and social and family norms influence their approach to managing these barriers.

**Conclusions:**

The key influences enabling or preventing optimal infant oral care identified in this study lay the foundation for interventions to target these behaviours. To encourage a timely first dental visit, parents need consistent messaging from dental and other health professionals. To encourage twice daily toothbrushing, parents need more support in managing their child’s behaviour and competing priorities.

**Supplementary Information:**

The online version contains supplementary material available at 10.1186/s12889-025-22487-9.

## Background

Dental caries (tooth decay) is one of the most prevalent childhood diseases in the world, affecting 621 million children [1]. Early childhood caries (ECC), defined as dental caries affecting the primary teeth of children under six years, has been reported to affect half of the world’s preschool children [2]. It is a leading cause of preventable hospitalisation in children under six years [3, 4]. In addition to pain, infection, and tooth loss caused by dental caries, ECC can adversely affect childhood growth, development and well-being during a critical developmental stage [5].

Dental caries is a complex disease caused by a change in the oral environment due to excessive sugar consumption that can lead to tooth destruction [6]. It is mostly preventable with twice daily toothbrushing with fluoridated toothpaste and limiting dietary sugar intake. These behaviours require support from parents and carers of young children. These behaviours, alongside timely dental visits, are considered optimal infant oral care.

As dietary and oral hygiene habits are established early in life and teeth are susceptible to dental caries as soon as they first erupt into the oral cavity, prevention needs to commence in infancy. As such, international guidelines recommend that toothbrushing commences when the first tooth erupts at approximately 6 months of age and professional dental check-ups occur by 12 months [7]. However, in Australia, toothbrushing and dental assessments often do not take place at the recommended ages [8].

Caregiver behaviours are critical to optimal infant and young child oral care, yet caregivers experience challenges to achieving this. A 2021 systematic review identified commonly reported barriers to toothbrushing, including lack of parental oral health knowledge, low confidence and social support, as well as misinformed beliefs, time constraints, and difficulty dealing with their child’s uncooperative behaviours [9]. Caregivers in the United Kingdom and Australia have reported that they require more support to adopt oral hygiene practices for their children, particularly relating to children’s resistant behaviour during toothbrushing [10, 11]. In an Australian study, commonly reported reasons for delayed dental visits include cost and beliefs that children are too young, have healthy teeth or will fear the dentist [12]. A study conducted in the United Kingdom revealed that managing children’s behaviour and environmental influences on family life were important in shaping parent habits [13].

Understanding the barriers and enablers (determinants) to adopting preventive oral health care behaviours is essential to inform the development of effective, evidence-based interventions and oral health promotion resources targeted towards parents and caregivers. Theoretical frameworks can offer meaningful and structured approach to understanding behavioural determinants. For example, the Theoretical Domains Framework (TDF) combines common elements of behaviour change theories into a single independently validated framework to evaluate factors that can either hinder or facilitate behaviour change [14]. A 2021 review of adoption of the TDF in oral and dental research found that studies from outside of Europe and North America, particularly relating to parent-supervised tooth brushing and first dental visit, are still lacking [15].

This study aimed to identify the barriers and enablers to early toothbrushing and dental visits for infants and young children faced by parents and caregivers in Australia. The findings of this study were planned to inform the development of behaviour change resources to support parents and caregivers to adopt optimal infant and young child oral care practices.

## Methods

### Study design

This study employed qualitative interviews with parents and caregivers of young children in Australia. Guided by a realist ontology [16], the research treated participants’ accounts as credible reflections of their experiences to understand the barriers and enablers related to two key behaviours: (1) the timing of children’s first dental visit and (2) twice-daily toothbrushing. The semi structured interviews and analysis were guided by the Theoretical Domains Framework (TDF).

### Participants

Eligible participants had to be: (i) parents or caregivers of a child under the age of 36 months in their care, (ii) residing in Australia, (iii) aged over 18 years, (iv) not participating in a related child oral health study conducted by the investigators and (v) able to communicate in English and willing to provide consent and participate. To yield a sample population that was representative of the Australian population, participants were purposively selected based on demographic characteristics including gender, highest education level, age, and marital status.

### Participant recruitment

**Fig. 1:**
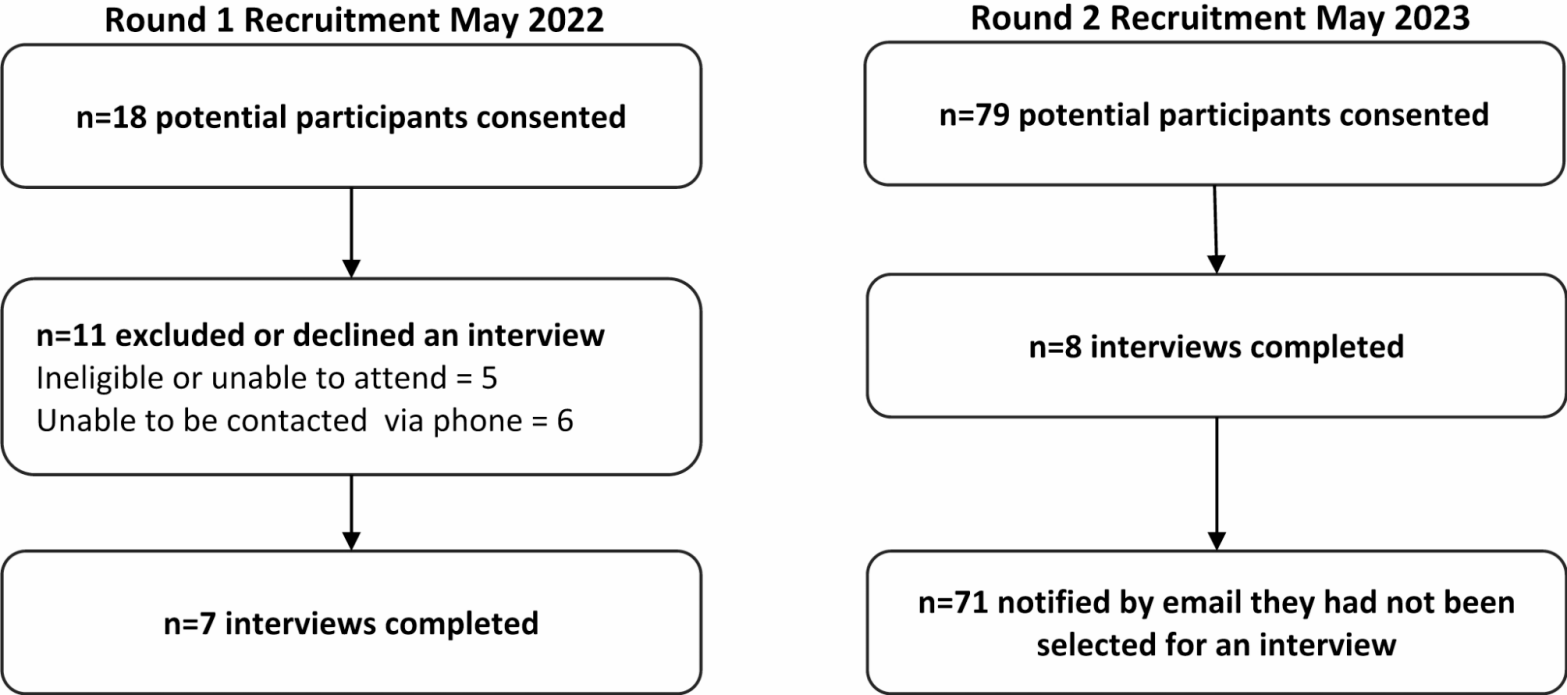
Recruitment Flowchart

Parents and caregivers were recruited via social media advertising and then subsequently contacted to schedule an interview. An initial round of recruitment in May 2022 was advertised through the social media accounts of the lead author’s research institute (MCRI) via posts that are viewed by followers of these accounts. This first round of recruitment yielded 18 expressions of interest with 7 participants completing interviews. The second round of recruitment was conducted in March 2023 and used the same social media post and platforms, additionally using paid advertising to reach people who were not followers of these account, and particularly to target male parents and caregivers and people living in areas with higher rates of area-level disadvantage. The second round of recruitment yielded 79 expressions of interest with 8 participants completing interviews. Recruitment is further outlined in Fig. 1.

The online advertisements included a link and QR code to an online consent form and short survey to obtain demographic information used for sampling (Supplementary File 1). Due to difficulty in predicting the number of people that would express interest and the intention to purposively select a representative sample, potential participants in the second round of recruitment were informed that they may not be contacted for an interview. Interviews concluded when the team determined enough new insights were collected to address the research questions. In line with a realist perspective [16], the researchers prioritised theoretical sufficiency over full data saturation, gathering enough information to explain key factors influencing parents’ and caregivers’ experiences. The decision to conclude interviews was also influenced by time and capacity constraints [17]. After this time, parents and caregivers who had expressed interest but were not interviewed were notified via email and invited to join as participants or family partners in other related research.

### Data collection

The interviews followed a semi-structured interview guide that aligned with the study aim and was mapped onto the TDF (Supplementary Table 1). The interview guide was pilot-tested, reviewed and adapted iteratively by the research team who have expertise in public health, paediatric dentistry, and psychology.

All interviews were completed by a single researcher (MT) online via Zoom video conferencing software (Zoom Video Communications Inc, California, United States). MT is a female Doctor of Clinical Dentistry Student specialising in paediatric dentistry. MT undertook qualitative research training as part of this degree and was guided by co-investigators with considerable qualitative research experience (AC, KG-B, SM). No participants were known to the researchers prior to being interviewed.

All interviews were audio recorded and transcribed by a professional third-party service. Data was anonymised prior to analysis.

### Analysis

A framework analysis approach was used to analyse the data, following Richie and Spencer’s framework analysis guidance (1994).

Data was coded using an adapted version of the TDF. The TDF was adapted based on a literature review prior to commencement of analysis. Codebook domain definitions were created based on a literature search and minor changes were made to the original TDF to better suit the oral health context (Supplementary Table 2). For example, social/professional roles and responsibilities is defined in this project as ‘role of parent as a provider of oral care’ [13]. To enhance credibility, two researchers, MT (DCD, BDS) and AC (Cpsychol, PhD), coded the first four transcripts concurrently and independently. Discrepancies between the two examiners’ coding was discussed and decided amongst the team over a series of meetings. Coding definitions were iteratively refined as new data were analysed, ensuring a reflexive and collaborative approach to data interpretation.

The codebook included a clear definition of the domains, examples, rules for application and additional notes. The codebook combined the TDF domains ‘Intentions’ and ‘Goals’ as they often appeared in tandem during the interviews. Where key data was not captured by any of the TDF domains, additional categories were added to the codebook. This combination of deductive and inductive coding allowed for greater flexibility while coding to ensure a comprehensive and accurate reflection of the barriers and enablers reported by parents/caregivers was captured.

Data was mapped onto the corresponding TDF domain, summarised, then categorised as a barrier or enabler. From this data overarching themes with underpinning sub-themes were developed by the team to meaningfully categorise the key barriers and enablers. Regular meetings were held with the broader research team throughout the analysis process to confirm understanding of the data and resolve uncertainty in mapping data to the TDF matrix.

## Results

A total of 97 parents/caregivers completed the online consent form, with 15 subsequently taking part in interviews conducted between May 2022–2023. Demographics of all participants who completed the initial consent are outlined in Supplementary Table 3. The interview duration ranged from 27 to 50 min lasting an average time of 37 min. All participants were parents, and included 11 females, 3 males and one parent with unspecified gender. Participant demographics are detailed in Table 1. The participant’s name attributed to each quote is a pseudonym.

**Table 1:** Participant demographics

Characteristic	**Category**	**Frequency (** ***n*** ** = 15)**
**Gender**	Female	11
	Male	3
	Unspecified	1
**Participant’s age (years)**	18–24	1
	25–34	6
	35–44	8
**Marital status**	Single	1
	Married	8
	Living with partner	4
	Separated	2
Highest education level*	Completed high school	1
	Certificate	1
	Bachelor’s degree	4
	Master’s degree	2
Age of youngest child (months)	0–5	1
	6–11	2
	12–17	5
	18–23	3
	24–29	1
	30–36	3
Caring for how many children	1	7
	2	7
	3	0
	4	0
	5	1
Australian State or jurisdiction of residence	Australian Capital Territory (ACT)	2
	New South Wales (NSW)	1
	Northern Territory (NT)	0
	Queensland (QLD)	1
	South Australia (SA)	1
	Tasmania (TAS)	0
	Victoria (VIC)	9
	Western Australia (WA)	1
Rural, Remote and Metropolitan Area (RRMA) classification	Metropolitan zone (RRMA 1 & 2)	9
	Rural zone (RRMA 3–5)	5
	Remote zone (RRMA 6 & 7)	1

*Only 8 participants answered this question. This question was introduced after the first social media post.

All 14 TDF domains except *optimism* were represented in the data. The most frequently coded domains were *social influences*, *environmental context and resources*, and *knowledge*. These prominent domains suggest that parents’ decisions about early toothbrushing and dental visits were shaped significantly by their social environment, the availability of resources, and the information they receive. Parents appeared to weigh up immediate and long-term outcomes and adapt their strategies accordingly. Social norms could act as barriers but knowledge about the importance of oral health and *beliefs about consequences* of early hygiene habits could facilitate optimal oral health habits. Knowledge gaps were present related to parents’ general awareness of oral health and specific concerns related to their child. Emotion was a prominent domain in shaping parental behaviours, particularly as parents desired positive oral health experiences for their children.

Four themes were developed from the TDF coded data: (1) Conflict, (2) Family and social norms, (3) Wanting a positive oral health experience, and (4) Uncertainty. These themes, along with their associated subthemes, and TDF domains are presented in Table 2. In the following sections, each theme will be discussed in detail to explore how they serve as either barriers or enablers to oral health behaviours.

**Table 2:** Themes, subthemes, and theoretical domains framework (TDF) domains

Theme	Subthemes	Most prominent TDF domain(s) per subtheme^*^
Conflict	Managing conflict	• Behaviour regulation
	Avoiding a domino of negative outcomes	• Beliefs about consequences • Intentions and goals • Decision processes • Social role
	Competing priorities	• Intentions and goals • Decision processes
Family and social norms	Influence of own/family oral health experiences	• **Social influences** • **Environmental context and resources**
	Perceived importance of baby teeth	• **Social influences** • **Knowledge**
	Social perception of dental practitioners and visits	• **Social influences**
	Parents as peer-support	• **Social influences**
Wanting a positive oral health experience	A gentle approach to oral healthcare	• Emotion • Skills
	Rapport with dental practitioners	• Emotion • Reinforcement • **Environmental context and resources**
Uncertainty	Mixed messages	• Knowledge • Skills • Reinforcement • **Environmental context and resources**
	Uncertainty navigating health services	• **Knowledge** • Reinforcement • **Environmental context and resources**

^*^Bold font indicates the three most prominent domains found in the data

## Theme 1: conflict

This theme reflects the conflict parents faced as they navigated their parenting priorities to prevent negative outcomes, such as their child becoming distressed or experiencing poor long-term oral health. Parents managed their actions and weighed the potential consequences of their decisions when addressing their child’s oral health, seeking to avoid conflict and achieve positive outcomes.

### 1.1 Managing conflict in the face of resistance

In order to avoid conflict, some parents avoided brushing their child’s teeth when they experienced resistance from their child. This avoidance was motivated by wanting to maintain a positive relationship with their child and harmony not only in the short-term, but also long-term by trying to avoid building negative associations with toothbrushing and oral health care at large.

*“I would leave it up to him to brush his own teeth*,* because I didn’t want to have the fight and then make him hate it*,* and then have real big discourse around dentists and oral care”– Stephanie (mother)*.

Parents felt that assistance with managing their child’s resistance to toothbrushing, rather than instruction about toothbrushing itself, would facilitate optimal habits at home.

*“It’s not necessarily the actual action of tooth brushing*,* it’s more about assistance in managing their tantrums and emotions and things around trying new things or keeping up with things that they might not necessarily enjoy. Managing that and tooth brushing falls under that”– Ashley (mother)*.

### Avoiding a domino of negative outcomes

Parents were aware of potential negative outcomes that may result from a lack of optimal dental behaviours. Goal setting enabled optimal oral health behaviours by seeking to avoid the long-term impacts of poor oral health, such as the financial burden associated with dental treatment:

*“I don’t want them to rot and […] have lots of teeth problems and tooth ache and dental problems and dental bills in the future and everything”– Stacey (mother)*.

As such, the role of the parent as the provider of oral care was identified as an enabler to avoid negative outcomes:

*“Occasionally they’ll brush their own*,* but I do make sure […] that I do it at least once a day for both of them. I let them do it*,* maybe once a day*,* but always make sure that I do it once a day as well”– James (father)*.

In recognising his role as a parent in caring for his child’s teeth at least once a day, James believes that he will enable his children to avoid future complications whilst also giving them the agency to try it themselves.

### Competing priorities

Parents encountered challenges prioritising their child’s needs and parenting goals. At times, oral health behaviours conflicted with broader caregiving considerations and familial well-being. Oral health care that was perceived to conflict with a child’s immediate needs, such as sleep or nutrition, were more likely to be de-prioritised:

*“I’ve just about got him to sleep and then I realise that I didn’t brush his teeth*,* and his sleep is really tricky. He’s not a good sleeper so I’m just like I’ll do it in the morning”– Laura (mother)*.

Time constraints, especially in the morning, led to oral health being considered a lower priority and resulted in toothbrushing not being done:

He’s three and sometimes getting pants on is a half hour negotiation. And sometimes, yeah, it’s just literally you’re dressed, you’ve got to get to day care because Mum’s got to leave to go to work. So it’s just running out of time with the negotiation… we prioritise clothes.

- Rae (mother)

In summary, this theme underscores the complex balancing act parents perform when managing their child’s oral health alongside competing priorities.

## Theme 2: family and social norms

The theme ‘Family and social norms’ captured how parents’ behaviours regarding their child’s oral health were influenced by personal experiences, family and broader societal norms. This theme highlighted the impact of family dynamics, parental upbringing, and cultural expectations on oral health behaviours, illustrating how parents’ actions were shaped by both immediate social circles and wider social perceptions in managing their child’s oral health.

### Influence of own/family oral health experiences

Parental approaches to their children’s oral health behaviours were shaped by norms rooted in their own childhood experiences. Expectations established during their upbringing influenced how they supported practices like regular toothbrushing and early dental visits. Many parents recalled that in their own childhood experience habits like toothbrushing were consistently encouraged, and expected, and this approach was something they consciously carried forward into caring for their own children. This has been shown by Anna:

*“In my household it was that*,* no you have to brush teeth. […]*,* you have to brush your teeth before bed and in the morning and if you’d gone to a party and eaten sugary foods you brush your teeth again. So*,* I guess it’s partially the way we were raised has had such an impact” - Anna (mother)*.

In contrast, parents who had less positive oral health experiences in their own childhood had concerns about providing oral health care for their children:

*“especially like*,* I was a kid and I hated brushing my teeth*,* so I didn’t want to make it a really big thing”– Stephanie (mother)*.

This reflects how early personal experiences can shape parental attitudes and influence the degree of emphasis placed on oral health behaviours for their own children, aligning with broader family and social norms.

### Perceived importance of primary teeth

Parents’ views on the importance of primary teeth influenced the oral care they provided. Some felt less motivated to establish strict oral health routines, believing that primary teeth, being temporary, did not require the same level of care as permanent teeth. This perception often led to less active engagement with oral health care as the temporary nature of primary teeth reduced the perceived urgency. This was illustrated by James:

*“I’d be reluctant to invest too much in teeth that are going to be replaced anyway”– James (father)*.

In contrast, other parents viewed primary teeth as important and emphasised the need to establish healthy habits early in life. They discussed both short and long-term benefits in caring for their child’s teeth:

*“It’s funny because a lot of people are like*,* no*,* they’re not that important because they fall out anyway but I’m like*,* no*,* it sets up good habit hygiene and also the other teeth are there*,* you don’t want infection*,* you don’t want it to go down. So baby teeth to me it is*,* they are important*,* they’re all teeth*,* they’re all important”– Anna (mother)*.

Anna’s knowledge about the importance of oral health and her awareness of the impact of early hygiene habits overcame social norms to enable the establishment of healthy oral care routines from a young age.

### Social perception of dental practitioners and visits

Parents had negative perceptions of dental practitioners, often characterised by mistrust about dental practitioners’ motivations. This created a significant barrier to regular dental check-ups for their children and following their advice on optimal oral care for their children. This was evident in Jason’s hesitation to book a dental appointment due to concerns that the dentist would simply ‘take his money’:

*“If I went to a different website with a dentist and they’re saying ‘…*,* you’ve got to book in for your check-up’*,* I’m like ‘Yeah*,* because then you can take my money’”– Jason (father)*.

The perception of dental practitioners and dental clinics as frightening for children was another significant barrier to regular dental visits. Parents who held these views were often reluctant to schedule appointments, fearing that the experience would cause distress or anxiety for their child. Parents own negative perceptions and fears reinforced these concerns, as shown by Carol:

*“Also dentists are really*,* I don’t know*,* medical and a bit scary and my older sons have lots of anxiety about going in a dental van. You know*,* people are in masks and all these like yucky smelling stuff and it’s just being like something stressful that I wouldn’t want to put her through…”– Carol (mother)*.

Such perceptions underscore how social influences and norms around dental care can impact parents’ willingness to engage with oral health services for their children.

### Parents as peer-support

Social support, particularly conversations with friends and family, can act as a facilitator. Speaking with others who have faced similar challenges and successes provided parents with practical advice and reminders. As shown by Tegan who described how a conversation with a friend prompted her to take her child for a dental visit:

*“My friend said she was going to take her son to the dentist. And I thought ‘oh um it’d be good to like take her to one of my appointments’”– Tegan (mother)*.

While parents valued speaking with others who shared similar experiences, they also discussed how this could sometimes lead to misinformation, particularly through online platforms. Although social interactions often provided reminders and encouragements, some parents expressed concerns that the information shared in these contexts was not always accurate or aligned with professional recommendations:

*“Running it by the parents’ group as well was*,* actually it wasn’t particularly helpful because they had not been doing it properly either but it was good to talk to them about it.”– Emma (mother)*.

In summary, this theme illustrates how family norms, personal experiences, and social views shape parents’ approaches to their child’s oral health. Parental attitudes toward primary teeth, perceptions of dental practitioners, and shared experiences with others all play roles in shaping these practices, demonstrating that social norms can both facilitate and hinder optimal oral health behaviours in early childhood.

### Theme 3: wanting a positive oral health experience

The theme ‘Wanting a positive oral health experience’ captured how parents’ desire to create positive and enjoyable oral health experiences for their children shaped their approach to both toothbrushing and early dental visits. The theme highlights parents’ focus on building supportive, stress-free routines, illustrating how a positive outlook and emotional connection influenced their management of their child’s oral care.

### A gentle approach to oral healthcare

Parents described many strategies to overcome the potential resistance to toothbrushing and dental visits. This included using play and songs to create positive associations with dental experiences. This gentle approach aimed to establish a more enjoyable and anxiety-alleviating atmosphere, helping children feel more comfortable and engaged. This was shown by Louise who described working in small steps and ‘not forcing it’:

*“We did a lot of play […] I’ve done a very gentle introduction to brushing his teeth*,* so not forcing it. […] So trying to introduce a positive association with brushing his teeth […] he’s been quite happy to do it because we […] sing the Play School song”– Louise (mother)*.

Some parents reported on compromising on toothbrushing to prioritise the gentle approach. For example, some parents described how this may have caused delays in optimal behaviours:

*“So*,* we’re trying to keep it as positive as possible even if it means that we don’t get as good a clean on the teeth as we’d like”– Oliver (Father)*.

Positive associations were perceived as supporting long-term engagement with optimal oral health behaviours. However, this did mean that approximations to optimal oral health behaviours were performed rather than the officially recommended optimal oral health behaviours.

### Rapport with the dental practitioner

Parents described feeling comfortable and supported in seeking oral care advice when they had a strong relationship with their dental practitioner. This rapport increased their confidence in managing their children’s oral care highlighting the significant impact of interpersonal relationships on health-related behaviours.

*“If I had any concerns*,* I know that our dentist is lovely and if I was to go in*,* she would help me get some ideas and strategies”– Jo (mother)*.

In contrast, others highlighted how poor communication and an ‘aggressive’ approach from practitioners not only undermined their confidence but also generated feelings of anxiety and distrust. For instance, Anna described feeling frustrated, as though she was being dictated to:

*“I think you got to find the right fit in terms of the way they deliver the information […]. It was like almost aggressive about it*,* just going*,* ‘no*,* he can’t […]*,* after they brush their teeth*,* they eat for this long’”– Anna (mother)*.

This negative rapport illustrates how poor practitioner communication can create emotional barriers that discourage parents from seeking or trusting oral health advice. Overall, the theme highlights the emotional connections in shaping parents’ strategies for their children’s oral care whereby parents prioritised supportive, relaxed interactions to make oral health routines more appealing for their children and building trust with dental practitioners was valued.

## Theme 4: uncertainty

The theme of ‘Uncertainty’ captures the confusion parents experience regarding their child’s oral health care. This uncertainty encompasses various aspects, such as selecting appropriate oral care tools and determining the right time for the child’s first dental visit. This theme highlights the gap between parents’ general awareness of oral health and specific concerns related to their child.

### Mixed messages

Parents reported confusion stemming from contradictory information about oral health care. The variety of sources, ranging from trusted healthcare professionals to media and informal networks, often provided mixed advice, making it challenging for parents to identify the best practices for their child’s oral health. These mixed messages contributed to the uncertainty that complicated their decision-making processes.

For example, parents expressed confusion regarding the choice of toothpaste for young children and what is needed:

*“I feel like there’s a bit of a discussion about fluoride toothpaste versus non-fluoridated and when you should introduce toothpaste and when not to*,* […] I feel like some people are told not to introduce toothpaste until 2 and I think that’s—I don’t know*,* yeah*,* like I just don’t know […] like mixed messaging”– Clara (mother)*.

Parents also discussed how they received mixed messages from dental practitioners, which added to their confusion. Some practitioners recommended scheduling a child’s first dental visit by a certain age, while others suggested waiting longer. This inconsistency made parents unsure about when it was best to take their child to the dentist, complicating their efforts to establish optimal oral health behaviours:

*“I don’t know what age you’re supposed to take them and I had conflicting information looking that one up as well. They’re like*,* oh ‘they can go at one and a half or two to start getting them used to it’. When I took him in at three and a half*,* they’re [dentist] like*,* ‘oh this is early’*,* and I was like*,* well which is it?”– Anna (mother)*.

A lack of consistent messaging acted as a barrier to both twice daily toothbrushing and timely dental care.

### Uncertainty navigating health services

Parents reported confusion about dental visits in early childhood, particularly as information often related to specific schemes with limited and complex eligibility criteria.

*“I guess what probably would help would be that clarity on what– if there is dental checks covered for children*,* like all children not people that are just on some sort of government payment because then that would be the avenue of where you would go to seek advice and tips.” - Jane (mother)*.

Information that was useful for all children, rather than scheme-specific guidance may alleviate confusion and facilitate early first dental visits. However, in light of this confusion, parents noted the potential role of early childhood health professionals, such as general practitioners and maternal child health nurses for early oral health assessment. Parents were confident about the ability of these health professionals in screening their child’s oral health and providing referral for dental care.

“*I think it’s almost like a GP and maternal health nurse can actually sort of check briefly and you can tell if there’s decay and stuff on kid’s teeth.” - Renee (mother)*.

Lack of understanding the benefit of a visit to a dental practitioner was a barrier for early dental visits, with one parent, for example, describing how he would go to the doctor in the first instance to look at his child’s teeth before going to the dentist:

*“I wouldn’t see any particular reason to give […] her a dental appointment*,* unless maybe a doctor has looked at them and said that there was a reason to worry.”– James (father)*.

This reliance on the family doctor illustrates their need for clarity about navigating health services reflecting parents’ uncertainty about the best path for addressing their child’s oral health needs.

## Discussion

In this study exploring parents and caregivers’ barriers and enablers to their infants and young children’s toothbrushing and early dental visits, we found that parents were navigating complex decision making regarding oral health care for their children, driven by managing conflicts and avoiding negative consequences. Uncertainty and parents’ own experiences as well as broader social norms were critical influences that ultimately shaped this decision-making process.

The potential for children to feel distressed by early dental visits (distinct from the actual experience of it) is the main consideration behind decisions about early dental visits. However, there was broad variation in how this shaped behaviours due to interactions with parents’ own health behaviours, self-efficacy about oral health, and perceptions of dental practitioners. Parents who perceived value in early dental check-ups, particularly through positive perception of dental practitioners, were more inclined to provide early positive experiences for their children. A systematic review analysing oral health promotion approaches in dental practices reported that families were more likely to be receptive to advice from dental practitioners who were empathetic and acknowledged the barriers they face, and with those who demonstrated enthusiasm and sincerity when providing information [18]. However, uncertainty about the timing and benefits of early dental visits due to mixed messages and concerns about costs, together with lack of engagement with oral health by non-dental health services led to parents delaying dental visits. These findings are consistent with other international studies, including a US study that revealed that mixed messages from dentists can deter optimal dental care for young children [19]. Our findings support the need for clearer messaging about early dental visits including from dental practitioners, and involvement of early childhood professionals outside of the dental sector [18, 20]. However in order to meet the expectations of families, it is important that non-dental practitioners are adequately trained and supported with regard to oral health concerns for children [20, 21].

The main toothbrushing challenges parents face related to the opposition they frequently encounter in young children, which is exacerbated by competing priorities ranging from other health needs to time constraints. These findings are consistent with other studies in the area including a recent qualitative study of British parents [13]. Adopting the TDF highlighted the complex interactions across 14 behavioural domains, with social influences, environmental context and resources, and knowledge were the most frequently coded. For example, uncertainty about the timing and benefits of early dental visits was influenced by mixed messages and concerns about costs, while the relationship with dental professionals played a dual role in addressing both social influences and knowledge.

Using the TDF allowed us to categorise these findings in a theoretically and methodologically robust way, providing a universal language of behaviour change. This approach establishes a strong foundation for developing targeted interventions that address key determinants such as improving parents’ knowledge of when to visit the dentist through accessible resources, developing trusting relationships with dental professionals, and providing strategies to manage child resistance and competing demands. Interventions lacking a theoretical underpinning risk oversimplifying these behaviours; for instance, traditional oral health education may enhance knowledge but fail to address the broader, interconnected factors needed to support sustained, long-term behaviour change [11, 22].

Although this study employed multiple recruitment methods to include both parents and caregivers across a diverse background, all those who expressed interest in taking part in the study were parents. This outcome likely reflects the heightened caregiving responsibilities assumed by parents during the COVID-19 pandemic, compounded by the shift toward remote working arrangements for many families [23]. These contextual factors may have influenced not only caregiving dynamics but also how parents navigated competing priorities and accessed oral healthcare for their children. Consequently, the findings provide rich and contextually grounded insights into the experiences of parents and can be meaningfully transferred to similar demographic groups. However, the absence of other caregiver perspectives, such as grandparents, limits the diversity of caregiving experiences captured. Future research incorporating multiple family members and health professionals could enhance data source triangulation for a more comprehensive understanding. Additionally, comprehension of English was a requirement to take part in this study which limited the ability of some linguistically diverse families to contribute to the study findings.

## Conclusions

Barriers and enablers to optimal infant and young child oral care are multi-faceted and driven by knowledge, emotion, perceptions of the role of parents as caregivers and social and family norms. While traditional oral health interventions around toothbrushing tend to focus on increasing knowledge, this study highlights the important influence of external factors in supporting behaviour change. In particular, interventions ought to acknowledge competing health priorities and provide strategies for managing children’s behaviour. To encourage both recommended toothbrushing practices as well as a timely first dental visit, parents need consistent messaging from dental practitioners in addition to other early life health professionals.

## Electronic supplementary material

Below is the link to the electronic supplementary material.

Supplementary Material 1: Supplementary Table 1. Overcoming Barriers Interview Questions mapped to the Theoretical Domains Framework (TDF). Description: Interview guide questions./

Supplementary Material 2: Supplementary Table 2. Theoretical Domains Framework (TDF) Domain and Definitions. Description: Table of TDF domains and definitions

Supplementary Material 3: Supplementary Table 3. Demographics of potential participants excluded or declined an interview. Description: Demographics table of potential participants

Supplementary Material 4: Oral Health and Your Child– Interview Research Study. Description: Participant consent and demographic survey.

## Data Availability

The data analysed in this study are not publicly available due to the sensitive nature of the interviews and potential re-identification of interview participants but are available from the corresponding author on reasonable request.

## Abbreviations

ECC: Early childhood caries
TDF: Theoretical Domains Framework
