# Supplementary material for: Parents’ perceived barriers and enablers to providing optimal infant oral care
Source: BMC Public Health. 2025 Apr 5;25:1292. doi: 10.1186/s12889-025-22487-9 (PMC11972519; doi:10.1186/s12889-025-22487-9)
Supplement: Supplementary file 1 — Supplementary Material 1: Supplementary Table 1. Overcoming Barriers Interview Questions mapped to the Theoretical Domains Framework (TDF). Description: Interview guide questions./ [file 12889_2025_22487_MOESM1_ESM.docx]

**Supplementary Table 1.** Overcoming Barriers Interview Questions mapped to the Theoretical Domains Framework (TDF)

| **Domain** | **Definition** | **Toothbrushing/ dental care** | **FDV** |
| --- | --- | --- | --- |
| 1. Knowledge | Knowledge around toothbrushing (benefits, duration, frequency, supervision, toothpaste, type of brush) tooth decay, age of first dental visit, role of the dentist | Do you know when s/he will get their first tooth?  If you have a question about your baby’s teeth, who/what sources of information do you turn to?  How were you feeling about the amount of support or information about your baby’s first tooth?  Were you looking for information about his teeth or were any provided for you  What kinds of information?  Do you know what toothpaste to use? | When do you feel is the appropriate time to go to the dentist for the first dental check up?  What information do you think would be helpful for you prior to taking them to the dentist? |
| 1. Skills | Competence and ability to conduct toothbrushing, coping strategies at managing oral care, developing child’s skills | How did you go about looking after her teeth around that age?  What makes it hard to fit the toothbrushing in?    Is it a one or two-people job to get the brushing done? |  |
| 1. Social/Professional Role and Identity | Role of parent as provider of oral care | How many times do you think s/he should have their teeth brushed? |  |
| **Domain** | **Definition** | **Toothbrushing/ dental care** | **FDV** |
| 1. Beliefs about capabilities | Self-efficacy, perceived competence in conducting oral care, perceived control^5^ | Did you know what to do when you saw that first tooth?  How are you managing the toothbrushing for your baby?  How well do you feel s/he’s brushing?  How well do you feel you’re brushing? | What have you found made it easier or harder to take your child to the dentist? |
| 1. Optimism | The confidence that things will happen for the best or that desired goals will be attained | *Can be covered by other questions – responses may fall into the domain* |  |
| 1. Beliefs about consequences | Parent outcome expectations of not conducting oral care, parent perceived outcomes for toothbrushing, parent perceived outcomes for going or not going to the dentist, attitudes towards oral care | Why do you look after your child’s teeth? | What are your expectations of a FDV? |
| 1. Reinforcement | Using rewards/incentives, punishments, creating routines, modelling | Can you describe your current brushing routine for your child?  morning v night v weekend routine  Can you give me examples of what you use as an incentive?  Who usually does the brushing? Wider family influence (any sleepovers at grandparents etc – change routine?) |  |
| 1. Intentions and goals | Conscious decision to perform oral care in a certain way, target setting, commitment^7^ | When did you start introducing any brushing or cleaning of her teeth?  What prompted you to start brushing? | Have you taken your child to the dentist? If yes, please tell me about your experiences If no, can you tell me why that is? |
| 1. Memory, attention, and decision processes | Ability to retain information, ability to choose between alternatives (including navigating conflicting online information), remembering to brush ^7, 11^ | If you have a question about your baby’s teeth, who/what sources of information do you turn to?  Have you found the information helpful? | Choosing between dental practices – private v public  Why did they go where they went – how they decided. Previous experience influence future decision making?  Where would they go and why? |
| **Domain** | **Definition** | **Toothbrushing/ dental care** | **FDV** |
| 1. Environmental Context and Resources | Sources of oral health information, costs and financial considerations (private health insurance, CDBS), bedtime/sleepiness, demands on time (work/other children), location (different home, with another carer, proximity of dental services)^4,6 ,11, 12, 13,14, 15^ | Was this information sufficient for you?  Where do you normally find information?  What kinds of information? E.g what toothbrush/paste, how to manage behaviour, frequency of brushing  What would you like to get more information on (and through what metho: phone, f-t-f, website etc)?  When’s the best time to get advice?  Who should provide it? | Do you know where to access dental care?  Eligibility knowledge – expand on the above question (costs, access requirements etc.)  Experience of going for FDV – ask generally if more than one child |
| 1. Social influences | Social norms/pressures/media, other carer’s/children’s/parent’s beliefs and practices in oral care, learning/modelling, trust in health professionals^6, 10, 11^ | Can you tell me a bit about yourself and your child/children?  How were you feeling about the amount of support or information about your baby’s first tooth?  If you have a question about your baby’s teeth, who/what sources of information do you turn to? | Who goes to the dental visits? |
| 1. Emotion | Resilience, anxiety, stress, burn-out, fear (including parent and child fears of dentist) , indifference | Can you tell me more about your experience when your child got their first tooth?  How did you she go for her first toothbrushing experience? | How do you feel about going to the dentist?   - How do you feel about taking your child?   Where you concerned at all prior to that first dental visit? |
| **Domain** | **Definition** | **Toothbrushing/ dental care** | **FDV** |
| 1. Behavioural regulation | Self-monitoring, managing child’s behaviour, barriers and facilitators^3, 6, 7, 11,14^ | What have you found worked?  Want made it easier?  What are some strategies you use if XXX is not co-operating?  What are some difficulties you have encountered when looking after XXX’s teeth? (if morning hard – why morning in particular)  How does he cope with that? | How did your child cope during the dental visit?  Is there anything you do to make going to the dentist easier/more fun? |
