# Supplementary material for: Parents’ perceived barriers and enablers to providing optimal infant oral care
Source: BMC Public Health. 2025 Apr 5;25:1292. doi: 10.1186/s12889-025-22487-9 (PMC11972519; doi:10.1186/s12889-025-22487-9)
Supplement: Supplementary file 2 — Supplementary Material 2: Supplementary Table 2. Theoretical Domains Framework (TDF) Domain and Definitions. Description: Table of TDF domains and definitions [file 12889_2025_22487_MOESM2_ESM.docx]

| Supplementary Table 2. Theoretical Domains Framework (TDF) Domain and Definitions | |
| --- | --- |
| TDF Domain | **Definition** |
| Knowledge | Knowledge around toothbrushing (benefits, duration, frequency, supervision, toothpaste, type of brush) tooth decay, age of first dental visit, role of the dentist |
| Skills | Competence and ability to conduct toothbrushing, coping strategies at managing oral care, developing child’s |
| Social/Professional Role and Identity | Role of parent as provider of oral care |
| Beliefs about capabilities | Self-efficacy, perceived competence in conducting oral care, perceived control |
| Optimism | The confidence that things will happen for the best or that desired goals will be attained |
| Beliefs about consequences | Parent outcome expectations of not conducting oral care, parent perceived outcomes for toothbrushing, parent perceived outcomes for going or not going to the dentist, attitudes towards oral care |
| Reinforcement | Using rewards/incentives, punishments, creating routines, consistent information |
| Intentions and goals | Conscious decision to perform oral care in a certain way, target setting, commitment |
| Memory, attention, and decision processes | Ability to retain information, ability to choose between alternatives (including navigating conflicting online information), remembering to brush |
| Environmental context and resources | Sources of oral health information, costs and financial considerations (private health insurance, CDBS), bedtime/sleepiness, demands on time (work/other children), location (different home, with another carer, proximity of dental services) |
| Social influences | Social norms/pressures/media, other carer’s/children’s/parent’s beliefs and practices in oral care, learning, modelling, trust in health professionals |
| Emotion | Resilience, anxiety, stress, burn-out, fear (including parent and child fears of dentist) , indifference |
| Behavioural regulation | Self-monitoring, managing child’s behaviour, barriers and facilitators |
| Other |  |
