# Supplementary material for: Parents’ perceived barriers and enablers to providing optimal infant oral care
Source: BMC Public Health. 2025 Apr 5;25:1292. doi: 10.1186/s12889-025-22487-9 (PMC11972519; doi:10.1186/s12889-025-22487-9)
Supplement: Supplementary file 3 — Supplementary Material 3: Supplementary Table 3. Demographics of potential participants excluded or declined an interview. Description: Demographics table of potential participants [file 12889_2025_22487_MOESM3_ESM.docx]

**Supplementary Table 3**. Demographics of potential participants excluded or declined an interview

| Characteristic | Category | Frequency (n=81) |
| --- | --- | --- |
| Gender | Female | 79 |
|  | Male | 2 |
|  | Unspecified | 0 |
| Potential participant’s age (years) | 18-24 | 1 |
|  | 25-34 | 41 |
|  | 35-44 | 37 |
|  | 45-54 | 2 |
| Highest education level* | Diploma | 6 |
|  | Certificate | 7 |
|  | Bachelor’s degree | 36 |
|  | Master’s degree | 17 |
|  | Doctorate | 4 |
|  | Postdoctorate | 3 |
| Relationship to child | Mother | 79 |
|  | Father | 2 |
|  | Grandparent | 0 |
|  | Guardian | 0 |
|  | Other | 0 |
| *Only 73 participants answered this question. The question was added after the initial social media post. | | |
