# Supplementary material for: Parents’ perceived barriers and enablers to providing optimal infant oral care
Source: BMC Public Health. 2025 Apr 5;25:1292. doi: 10.1186/s12889-025-22487-9 (PMC11972519; doi:10.1186/s12889-025-22487-9)
Supplement: Supplementary file 4 — Supplementary Material 4: Oral Health and Your Child– Interview Research Study. Description: Participant consent and demographic survey. [file 12889_2025_22487_MOESM4_ESM.pdf]

Oral Health and Your Child - Interview Research Study

Thank-you for your interest in our research project. We are inviting you to take part in a study exploring your perceptions and experiences of your child's oral health care. In this project, we want to hear about your experiences when it comes to brushing your child's teeth and taking your child to the dentist.

If you are eligible to take part, we will provide detailed information about this research. If you feel like this study is right for you, and you would like to proceed, click next and it will take you to our consent page.

Contact details

If you have any questions about the project, you can contact the research team at overcomingbarriers@mcri.edu.au

Eligibility Screening

Are you aged 18 years or older?

◯ Yes
◯ No

Unfortunately, this study can only be provided to those aged at least 18 years. Thank you for your interest.

Do you currently live in Australia?

◯ Yes
◯ No

Unfortunately, this study can only be provided to people living in Australia. Thank you for your interest.

Do you have a child aged under 36 months in your care?

◯ Yes
◯ No

Unfortunately, this study is only relevant for those with children in this age range. Thank you for your interest.

Are you or your child currently enrolled in any other research projects?

◯ Yes
◯ No

Please name or describe the research project(s) you or your child are involved in

____

Your answers to the eligibility questions indicate that you are NOT eligible to participate. Please confirm that the above details are correct.

◯ Yes
◯ No

Please correct your answers and continue to the next page

Study Information

What the project is about

Having healthy baby teeth is very important. We would like to take some time to interview you and learn more about your experiences caring for your child's teeth and mouth, such as brushing teeth and visits to the dentist. This will help us identify practical strategies to support families with infants. We will also ask for your opinion on oral health information to find out what will work best for families.

Who can participate?

To be eligible for this study, you must be over 18 years of age, currently be a caregiver of a child under the age of 36 months and reside in Australia

What does participation involve?

Participation will involve a short survey and interview.

About the interview

The interview will take roughly 60 minutes to complete. An interviewer will ask you about your experiences and thoughts on brushing your child's teeth and taking your child to the dentist.

Interviews will be primarily conducted using video calls, therefore ideally you will have access to internet and use of a video-capable device. If you prefer or do not have access to video-calling, then the interview will be conducted over the phone. You may choose the option that is best for you. The interviews will be audio-recorded. We may also send you oral health information for your perusal along with a short survey for your opinion on how you feel about them.

Is participation voluntary and can I withdraw?

Participation is completely voluntary and you may withdraw at any time, without the need to give a reason.

Are there any benefits to my involvement?

Your participation will provide valuable insights to help researchers develop oral health care support for parents and caregivers of young children. You will receive a $30 gift card to thank you for your time and contribution at the conclusion of the study.

Your information and privacy protection

The information you provide us will remain confidential, stored securely, and will only be accessed by authorized researchers in this study. Data collected will be anonymized and may be published in future studies, scientific journals and reported at scientific meetings.

Your copy of this information

In addition to the information above, please read the attached plain language information and consent form before proceeding.

[Attachment: "Participant Information and Consent V2 01.02.2022.pdf"]

Study Consent

Study Number: 79551
Short Name of Project: Overcoming barriers to providing optimal oral care for infants
Version Number: 2.0
Version Date: 01/02/2022

- I have read this information statement and I understand its contents.
- I understand what I have to do to be involved in this project.
- I understand the risks I could face because of my involvement in this project.
- I voluntarily consent to take part in this research project and I am free to withdraw from the study at any time, without giving any reason.
- I have had an opportunity to ask questions about the project and I am satisfied with the answers I have received.
- I understand that this project has been approved by The Royal Children's Hospital Melbourne Human Research Ethics Committee. I understand that the project is required to be carried out in line with the National Statement on Ethical Conduct in Human Research (2007).
- I understand I will receive a copy of this Information Statement and Consent Form.

I have read and understood the above information and agree to participate in this research	◯ I do<br>◯ I do not
Date of participant consent	__________________________
Optional consent 1<br><br>I consent to the storage of the interview findings for use in ethically approved research, broadly related to dental and oral health<br><br>Your response to the optional consent will not affect your overall involvement with the study	◯ I do<br>◯ I do not
Optional consent 2<br><br>I consent to being contacted about future research<br><br>Your response to the optional consent will not affect your overall involvement with the study	◯ I do<br>◯ I do not

Thank you for agreeing to participate in this research study!

We now have a short survey, and a place for your information so that we can get in touch with you for the interviews.

About you and contact information

Please fill in your details below

First name Last name

______ ______

Email Phone number

______ ______

Postcode Age

______ ______

Please select which of the below describes your relationship with the child aged under 36 months in your care	◯ mother<br>◯ father<br>◯ grandparent<br>◯ guardian<br>◯ other
Please specify	__________________________________
With which gender do you most identify?	◯ female<br>◯ male<br>◯ indeterminate<br>◯ intersex<br>◯ unspecified<br>◯ other
Please specify	__________________________________
Marital status	◯ single<br>◯ married<br>◯ living with partner<br>◯ separated<br>◯ divorced<br>◯ never married<br>◯ other
Please specify	__________________________________
Highest education level	◯ Did not complete highschool<br>◯ Completed highschool<br>◯ Certificate<br>◯ Diploma<br>◯ Bachelor's Degree<br>◯ Master's Degree<br>◯ Doctorate<br>◯ Postdoctorate
How many children are in your care?	◯ 1<br>◯ 2<br>◯ 3<br>◯ 4<br>◯ 5<br>◯ 6+
What is the age of your youngest child in months?	__________________________________

When is the best time to contact you?<br>please only select one	◯ 9am-12pm<br>◯ 12pm-3pm<br>◯ 3pm-6pm
Do you have access to internet and a device that is zoom compatible?<br><br>Zoom is a free video software that you can access on your web browser or by downloading the app on either a phone or computer	◯ Yes<br>◯ No

Please note that all interviews will be conducted over zoom. Not having access to a zoom compatible device may exclude you from being eligible to participate in this study.
